# Network-Based Inference Methods for Drug Repositioning

**DOI:** 10.1155/2015/130620

**Published:** 2015-04-12

**Authors:** Hailin Chen, Heng Zhang, Zuping Zhang, Yiqin Cao, Wenliang Tang

**Affiliations:** ^1^School of Software, East China Jiaotong University, Nanchang 330013, China; ^2^School of Information Engineering, East China Jiaotong University, Nanchang 330013, China; ^3^School of Information Science and Engineering, Central South University, Changsha 410083, China

## Abstract

Mining potential drug-disease associations can speed up drug repositioning for pharmaceutical companies. Previous computational strategies focused on prior biological information for association inference. However, such information may not be comprehensively available and may contain errors. Different from previous research, two inference methods, *ProbS* and *HeatS*, were introduced in this paper to predict direct drug-disease associations based only on the basic network topology measure. Bipartite network topology was used to prioritize the potentially indicated diseases for a drug. Experimental results showed that both methods can receive reliable prediction performance and achieve AUC values of 0.9192 and 0.9079, respectively. Case studies on real drugs indicated that some of the strongly predicted associations were confirmed by results in the Comparative Toxicogenomics Database (CTD). Finally, a comprehensive prediction of drug-disease associations enables us to suggest many new drug indications for further studies.

## 1. Introduction

Drug discovery is a costly and time-consuming process. Previous research reported that it takes around 15 years and $800 million to $1 billion for pharmaceutical companies to develop a new drug and bring it to market [1, 2]. Although such huge amount of time and money has been put into this industry, only a relatively small number (~20) of new drugs known as new molecular entities (NMEs) are approved by US Food and Drug Administration (FDA) each year (http://www.fda.gov/Drugs/DevelopmentApprovalProcess/HowDrugsareDevelopedandApproved/DrugandBiologicApprovalReports/default.htm). Even so, there is a declining trend in annual drug introductions and the most significant cause of this productivity decline has been the decrease in development survival rates, especially the attrition rate in Phase II trials [3]. It was estimated that Phase II survival in drug development has dropped from near 50% to less than 30% [4, 5].

As the concept of “one drug, multiple targets” [6] is now widely recognized, drug repositioning or repurposing (i.e., finding a new use for an existing drug) [7] has been proposed to provide a solution to the above problems faced by pharmaceutical companies. For example,* Rituxan* was originally indicated for non-Hodgkin's Lymphoma and it was later approved for chronic lymphocytic leukemia and rheumatoid arthritis. Since drug repositioning can benefit not only pharmaceutical companies but also patients, various efforts, including traditionally blind screening methods of chemical libraries against specific cell lines [8] or cellular organisms [9, 10], and serial testing of animal models [11], have been made to search new indications for existing drugs. Meanwhile, to reduce cost and time of* in vivo* and* in vitro* experiments, many computational methods have been published for drug repositioning [12–22]. These methods can be classified as “drug based” or “disease based” and they mainly take similarity measures (chemical similarity, molecular activity similarity, or side effect similarity), molecular docking, or shared molecular pathology to reveal potential repurposing opportunities [23]. New drug targets, mainly proteins, genes, or pathways, are predicted by these methods for further drug repositioning. Applications and limitations coexist in these methods and comprehensive summaries of recent advancement of computational drug repositioning are available in the two reviews [23, 24].

More recently, several computational strategies have been proposed to predict direct drug-disease associations for drug repositioning. The Comparative Toxicogenomics Database (CTD; http://ctdbase.org/) [25] is a comprehensive and publicly available database which inferred chemical-disease associations by integrating chemical-gene interactions and gene-disease relationships received manually from published literature. Chiang and Butte [26] implemented a network-based, guilt-by-association method (GBA) for drug-disease association prediction and novel drug use suggestions were made based on shared treatment profile from disease pairs. This repositioning strategy is limited by the complex relationships between drugs and diseases as many drugs are indicated as palliative treatments for diseases, like cancers [23]. Based on the observation that similar drugs are indicated for similar diseases, Gottlieb et al. [27] developed a novel algorithm, PREDICT, to infer potential drug-disease associations and predict new drug indications. Multiple drug-drug and disease-disease similarity measures were integrated into their method and excellent prediction accuracy can be obtained on cross validation. However, negative samples were needed in their model to implement the prediction procedure. Experimentally verified negative drug-disease associations are not available due to lack of research value. A comodule (important gene modules shared by both drugs and diseases) method was proposed by Zhao and Li [28] to understand how drugs and diseases are associated in the molecular level. Applying the method to simulations and real data demonstrated that it was able to identify new drug-disease associations and highlight their molecular basis. Huang et al. [29] designed a network propagation model and exploited existing chemical, genomic, and disease phenotype data to infer drug-protein/gene-disease phenotype relationships, in which genes with similar functional modules were related to not only drugs but also the disease phenotype. Daminelli et al. [30] integrated structural and chemical data to build a drug-target-disease network and mined the network for network motifs of bi-cliques where every drug is linked to every target and disease. Links from drugs to diseases were predicted by completing the incomplete bicliques. Ye et al. [31] collected known drug target information and disease pathway profile to develop a method for evaluating the relationships between drugs and a specific disease. Emig et al. [32] integrated disease gene expression signatures, drug targets, disease information, and molecular interaction network to prioritize drug targets for drug repositioning.

Computational inference methods are important ways to choose the most promising drug-disease associations for further drug repositioning. Current computational methods need biologically relevant information, including negative association results, similarity measures, or gene expression profiles, to assist drug-disease association prediction. Such additional information may not be easily available or may contain errors [23]. Different from previous research, in this paper, two inference methods,* ProbS* and* HeatS*, were introduced to predict direct drug-disease associations based only on the basic network topology measure. We formulated the problem as recommending preferable diseases for drugs. Network topology was used to prioritize the potentially targeted diseases for drugs. We tested the two methods on an experimentally verified dataset with leave-one-out cross validation for performance evaluation. Experimental results showed that both methods can receive reliable prediction performance and achieve AUC values of 0.9192 and 0.9079, respectively. The method* ProbS* with a better performance was selected for potential drug-disease association prediction. Case studies demonstrated that some of the strongly predicted associations are confirmed by the publicly available database CTD [25], which indicated the practical applications of the method* ProbS* in a real environment. Finally, plenty of predicted drug-disease associations were publicly released for future drug repositioning. It is expected that these methods will provide help to facilitate further research on drug repositioning.

## 2. Materials and Methods

### 2.1. Dataset

Experimentally confirmed drug-disease associations were downloaded from the supplementary material of [27]. At the time of the paper [27] was written, Gottlieb et al. collected 1933 associations between 593 drugs taken from DrugBank [34] and 313 diseases listed in the Online Mendelian Inheritance in Man (OMIM) database [35]. This set of known drug-disease associations is regarded as the “gold standard” data and is used for evaluating the performance of our introduced methods in the following cross validation experiments as well as training data in the comprehensive association prediction.

We denote the drug set as *D* = {*d*_1_, *d*_2_,…, *d*_*m*_} and the disease set as *P* = {*p*_1_, *p*_2_,…, *p*_*n*_}. The drug-disease associations can be described as a bipartite DP graph *G*(*D*, *P*, *E*), where *E* = {*e*_*ij*_ : *d*_*i*_ ∈ *D*, *p*_*j*_ ∈ *P*}. An edge is drawn between the drug *d*_*i*_ and the disease *p*_*j*_ if there exists an association between them. The DP bipartite network can be presented by an *n* × *m* adjacent matrix {*a*_*ij*_}, where *a*_*ij*_ = 1 if *p*_*i*_ and *d*_*j*_ is linked, while all other unknown drug-disease pairs are labeled as 0 to indicate that their associations need to be predicted.

### 2.2. Method Description

The methods* ProbS* and* HeatS* we applied in this paper are based on the recommendation techniques developed by Zhou et al. [33, 36]. We formulated our problem as recommending diseases for a given drug by mining data on the drug-disease bipartite network properties.

Given the drug-disease bipartite network defined above,* ProbS* works by assigning diseases an initial resource denoted by the vector *f*, and *f*(*p*_*i*_) is the initial resource allocated to the *i*th disease in the disease set *P*. The initial resource of an associated disease is set to be 1 in our study; otherwise it is set to be 0. In the first step, all the resource in the disease set *P* flows to the drug set *D* according to

(1) $$\begin{array}{c} f\left(d_{l}\right)=\overset{n}{\underset{i=1}{\sum }}\frac{a_{il}f\left(p_{i}\right)}{k\left(p_{i}\right)}, \end{array}$$

where *k*(*p*_*i*_) is the degree of node *p*_*i*_. Subsequently, all the resource in the drug set *D* returns similarly back to the disease set *P*. The final resource allocated to *p*_*i*_ is calculated as

(2) $$\begin{array}{c} f^{\prime }\left(p_{i}\right)=\overset{m}{\underset{l=1}{\sum }}\frac{a_{il}f\left(d_{l}\right)}{k\left(d_{l}\right)}=\overset{m}{\underset{l=1}{\sum }}\frac{a_{il}}{k\left(d_{l}\right)}\overset{n}{\underset{j=1}{\sum }}\frac{a_{jl}f\left(p_{j}\right)}{k\left(p_{j}\right)}. \end{array}$$

The final recommendation list of candidate diseases is sorted according to the results of (2) in a descending order. The disease(s) with the highest score(s) is chosen as the new indication(s) for the corresponding drug.

While for the method* HeatS* [36], the final resource allocated to *p*_*i*_ is calculated as

(3) $$\begin{array}{c} f^{\prime }\left(p_{i}\right)=\overset{m}{\underset{l=1}{\sum }}\frac{a_{il}}{k\left(p_{i}\right)}\overset{n}{\underset{j=1}{\sum }}\frac{a_{jl}f\left(p_{j}\right)}{k\left(d_{l}\right)}. \end{array}$$

**Figure 1:**
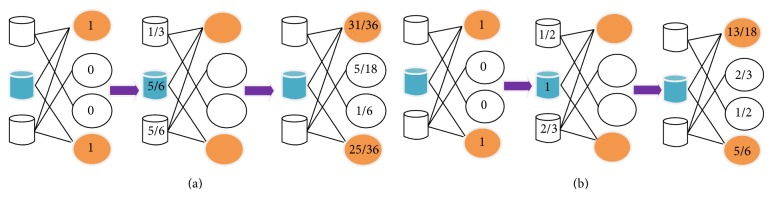
The flowchart of the two methods* ProbS* (a) and* HeatS* (b). The cylinder objects and the ellipse objects mean drugs and diseases, respectively. This figure is inspired by [33].

Comparison of the main pipeline of the two methods is illustrated in Figure 1.

## 3. Results

### 3.1. Construction and Characteristics of the Drug-Disease Association Network

**Figure 2:**
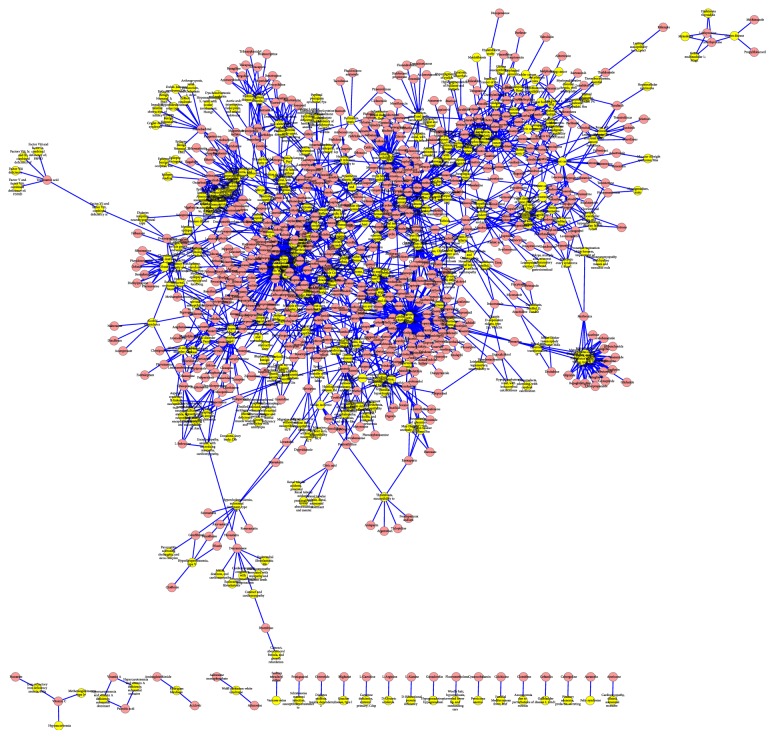
Drug-disease association network. Red rectangles and yellow rectangles indicate drugs and diseases, respectively. The bipartite network is generated by using 1933 experimentally verified associations between drugs and diseases. This network is prepared by Cytoscape (http://www.cytoscape.org/).

In this study, we first focused on the verified drug-disease associations. There were 1933 experimentally confirmed drug-disease associations, including 593 drugs and 313 diseases, on our benchmark dataset. We used the 1933 associations to generate a bipartite graph (Figure 2). In the bipartite graph, the heterogeneous nodes correspond to either drugs or diseases, and edges correspond to associations between them. An edge is placed between a drug node and a disease node if the drug is known to have an association with the disease. It can be observed that drugs tend to bind known diseases, forming highly interconnected subnetworks.

**Figure 3:**
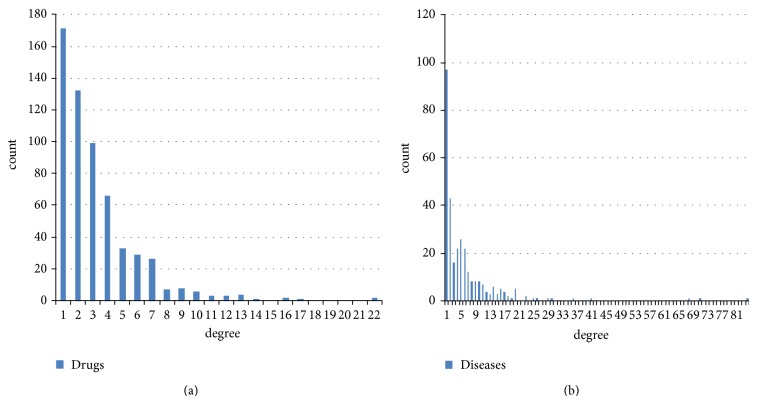
Degree distributions of drugs and diseases in the drug-disease network. (a) shows the histograms of the degree distributions of drugs. (b) shows the histograms of the degree distributions of diseases.

Figure 3 gives the degree distributions of drugs and diseases in the drug-disease network. We found that more than 70% (422/593) of the drugs were associated with at least two diseases, and approximately 66% (206/313) diseases were associated with two or more drugs. The average number of associated diseases for each drug is 3.26 and the average number of associated drugs for each disease is 6.18. More details are available in Figure 3 and Table 1.

### 3.2. Leave-One-Out Cross Validation for Performance Evaluation

To assess the comparative performance of both methods in predicting new indications for existing drugs, we perform leave-one-out cross validation experiments on the benchmark dataset. For a given drug *d*, each known associated disease was left out once in turn as test disease, whose initial resource was set to be 0, and the candidate disease set consisted of all the diseases which had no evidence to show their associations with the drug *d*. The entire associations were prioritized according to the scores derived from the two methods.

We calculated the sensitivity and specificity for each threshold. Sensitivity refers to the percentage of the associations whose ranking is higher than a given threshold, namely, the ratio of the successfully predicted experimentally verified drug-disease associations to the total experimentally verified drug-disease associations. Specificity refers to the percentage of associations that are below the threshold. Receiver-operating characteristics (ROC) curves were plotted by varying the threshold, and the values of area under curves (AUC) were calculated. When the two methods,* ProbS* and* HeatS*, were tested on the 1933 experimentally verified drug-disease associations in the framework of leave-one-out cross validation, reliable AUC values of 0.9192 and 0.9079 were received, respectively. To be instructive, we also provided AUC values for each drug received by leave-one-out cross validation (see Supplementary Material S1 available online at http://dx.doi.org/10.1155/2015/130620 for the method* ProbS* and S2 for* HeatS*).

We also calculated the positions of preferably drug-disease associations ranked on the recommendation list for the two methods. For instance, if there are 100 candidate diseases for a drug *d* and an associated disease *m* is ranked 5th in the candidate diseases, we say the position of the disease *m* is 5/100, denoted by 〈*P*〉 = 0.05. A good algorithm is expected to give a small 〈*P*〉. Therefore, we used the average value of the position 〈*P*〉 over all diseases in the candidate set to compare the algorithmic accuracy. The average values of the position 〈*P*〉 for* ProbS* and* HeatS* were 0.0319 and 0.0434, respectively.

The reliable performance suggested that both the two methods can recover the confirmed drug-disease associations and therefore has the potential to predict new drug-disease associations for drug repositioning.

### 3.3. Comparison with Other Methods

Recently, several computational models, which were based on different data features, have been proposed for drug-disease association prediction. Some information, like protein-protein interactions adopted in [29], has a high rate of false-positive and false-negative results, which will influence experimental results. The most recent study related with our work is the computational model proposed by Gottlieb et al. [27], which was based on the observation that similar drugs are indicated for similar diseases. Excellent prediction performance can be received by this method. However, one limitation of this model is that negative samples were needed for association prediction. Our method is based on the experimentally verified drug-disease associations and does not make use of any prior biological knowledge.

### 3.4. Case Studies

To further explain the inference power of the method* ProbS* on drug-disease association prediction, we conducted case studies on three drugs (*Felodipine*,* Aspirin,* and* Tamoxifen*). The whole candidate diseases were ranked according to the method. We manually checked the top 10 predicted associations and confirmed that 4, 6, and 6 associations (Tables 2 [Table tab3]–4) are now annotated in the CTD (http://ctdbase.org/) [25] database. We take these as a strong evidence to support the practical application of the method* ProbS*. Note that the predicted associations that are not yet reported may also exist in reality and they provide opportunities for drug repositioning.

### 3.5. Comprehensive Prediction of Drug-Disease Associations

After comprehensively confirming the accuracy of both methods, we chose* ProbS*, which showed a better performance, to further predict novel drug-disease associations for future drug repositioning. In this scenario, we trained* ProbS* with all the known drug-disease associations on the benchmark dataset. For all the 593 drugs, we ranked and selected the top 10 predicted diseases for drug repositioning (Supplementary Material S3). We believe that these predicted associations would benefit drug research.

## 4. Discussion

Searching novel drug-disease associations is a critical step in drug repositioning. Computational methods for drug-disease association prediction have received numerous interests and current computational strategies depend on prior biological information, including gene expression profiles, protein-protein interactions (PPI), or chemical structures. Such information may not be widely available and some may contain errors.

In this paper, two network topology-based inference methods were introduced to predict potential drug-disease associations. The essential difference between them is the resource-allocation strategies they applied. Even though both methods do not use any prior biological knowledge, experimental results showed that reliable prediction performance can be achieved by the two methods. Moreover both the two methods are easy to be implemented and they have a low time complexity of (*ο*(*n* ^2^ *m*)).

Despite the encouraging results produced by the two methods, some limitations should be noted. First, as only network topology is applied, both the two methods cannot work for drugs without any known drug-disease associations. To predict drug-disease associations for novel drugs, reliable similarity measure needs to be taken into consideration. Meanwhile, the predicted results may be biased as our methods depend heavily on existing drug-disease associations for predictions and our current knowledge about drug-disease associations is far from complete. Therefore, the performance of the methods could be improved by integrating more verified drug-disease associations.

## Supplementary Material

Supplementary Material S1: AUC values for each drug received by leave-one-out cross-validation when *ProbS* was applied.
Supplementary Material S2: AUC values for each drug received by leave-one-out cross-validation when *HeatS* was applied.
Supplementary Material S3: The top 10 predicted associations between each drug and diseases.

## Figures and Tables

**Table 1:** Statistics of the validated drug-disease association network.

Number of drugs	Number of diseases	Number of drug-disease associations	Average degree of drugs	Average degree of diseases	Sparsity
593	313	1933	3.26	6.18	0.0104

**Table 2:** The newly confirmed drug-disease associations in the top 10 predicted results of the drug *Felodipine*.

Drug	Disease	Rank	Status
*Felodipine *	Hypoparathyroidism, sensorineural deafness, and renal disease	1	Confirmed
*Felodipine *	Hypertension, essential	2	Confirmed
*Felodipine *	Hydrops-ectopic calcification-moth-eaten skeletal dysplasia	3	
*Felodipine *	Enteropathy, familial, with villous edema and immunoglobulin G2 deficiency	4	
*Felodipine *	Preeclampsia/eclampsia 1;* Pee1 *	5	
*Felodipine *	Insensitivity to pain with hyperplastic myelinopathy	6	
*Felodipine *	Glaucoma 1, open angle, A;* Glc1A *	7	
*Felodipine *	Acanthosis nigricans with muscle cramps and acral enlargement	8	
*Felodipine *	Atrial fibrillation, familial, 3;* Atfb3 *	9	Confirmed
*Felodipine *	Prostatic hyperplasia, benign;* Bph *	10	Confirmed

**Table 3 tab3:** The newly confirmed drug-disease associations in the top 10 predicted results of the drug *Aspirin*.

Drug	Disease	Rank	Status
*Aspirin *	Mitochondrial myopathy, encephalopathy, lactic acidosis, and stroke-like	1	Confirmed
*Aspirin *	Exostoses with anetodermia and brachydactyly, Type E	2	
*Aspirin *	Atrial fibrillation, familial, 1;* Atfb1 *	3	Confirmed
*Aspirin *	Atrial fibrillation, familial, 3;* Atfb3 *	4	Confirmed
*Aspirin *	Neuropathy, hereditary sensory and autonomic, Type I, with cough and gastroesophageal reflux	5	
*Aspirin *	Exostoses of heel	6	
*Aspirin *	Motor neuropathy, peripheral, with dysautonomia	7	
*Aspirin *	Migraine, familial typical, susceptibility to, 2	8	Confirmed
*Aspirin *	Myasthenia gravis;* Mg *	9	Confirmed
*Aspirin *	Coronary artery disease, autosomal dominant, 1;* Adcad1 *	10	Confirmed

**Table 4:** The newly confirmed drug-disease associations in the top 10 predicted results of the drug *Tamoxifen*.

Drug	Disease	Rank	Status
*Tamoxifen *	Mismatch repair cancer syndrome	1	
*Tamoxifen *	Chorioretinal dystrophy, spinocerebellar ataxia, and hypogonadotropic	2	
*Tamoxifen *	Prostate cancer	3	Confirmed
*Tamoxifen *	Acroosteolysis with osteoporosis and changes in skull and mandible	4	
*Tamoxifen *	Hypogonadism, male	5	Confirmed
*Tamoxifen *	Gastric cancer	6	Confirmed
*Tamoxifen *	Renal cell carcinoma, nonpapillary; Rcc	7	Confirmed
*Tamoxifen *	Kaposi sarcoma	8	Confirmed
*Tamoxifen *	Uterine anomalies	9	
*Tamoxifen *	Osteoporosis	10	Confirmed
